# Journey to the east: the oldest tetrapod fauna of east Pangea in early Permian

**DOI:** 10.1093/nsr/nwae249

**Published:** 2024-07-23

**Authors:** Jianye Chen, Jun Liu

**Affiliations:** Institute of Vertebrate Paleontology and Paleoanthropology, Chinese Academy of Sciences, China; Department of Earth Sciences, University of Hong Kong, China; Institute of Vertebrate Paleontology and Paleoanthropology, Chinese Academy of Sciences, China; College of Earth and Planetary Sciences, University of Chinese Academy of Sciences, China

## Abstract

New fossil footprints from Beijing shows that four-foot animals already roamed the east side of supercontinent Pangea ~300 million years ago, proving land connections between North China Block and Pangea.

The amalgamation of Pangea during the late Paleozoic is a major tectonic event in Earth history [1], but its process and timing is still not well understood, especially among its eastern margin. Two competing hypotheses exist on the collision time between the North China Block (NCB) and Pangea. In the ‘Early Collision’ hypothesis, NCB had been connected with the Tarim Block by the Devonian period, resulting in a completely connected NCB-Pangea unity by early Permian [2,3]. Alternatively, in the ‘Late Collision’ hypothesis, although Baltica-Kazakhstania-Tarim became connected by the Late Carboniferous or early Permian, NCB and Tarim Block remain separated until late Permian [e.g. 4,5], based primarily on different geological and paleomagnetic data between the Tarim Block and NCB [5]. The controversy resulted in different paleobiogeography reconstructions during the Permian, some with a united NCB-Pangea by early Permian and some with a sea still separating NCB and Pangea in late Permian [e.g. 4]. This in turn alters our views in modelling paleoclimate and evolution during the late Paleozoic [6–9].

Ever since the start of the continental drift theory, distribution of fossils was used to provide evidence for land connections. A textbook example is how similar fossils (e.g. *Mesosaurus, Glossopteris* and *Lystrosaurus*) found from across the southern continents (Africa, South America, Antarctica and India) prove the assembly of Gondwana during the Permian-Triassic [10]. Among all fossils, distribution of faunas across continents provides the strongest evidence for land connection, because rafting, swimming or other ways to cross the sea are highly unlikely to occur simultaneously by different animals of variable sizes in the same fauna [6].

Recently we discovered a new early Permian tetrapod footprint assemblage, the Mentougou Ichno-fauna, from the Taiyuan Formation (Asselian, 298 Ma) of Beijing, China (Fig. 1; Fig. S1–S17). These footprints were the earliest record of terrestrial tetrapods in eastern Pangea. A total of six slabs were cataloged (IVPP V31904–31909), each with one or more footprints of variable qualities (Fig. 1a–m, Fig. S1–S16, Supple. Data). Both anamniote and amniote footprints can be identified. Most identifiable footprints belong to *Limnopus*, probably produced by temnospondyls (Fig. 1a–d, i, j; Fig. S1–S8). One footprint is probably produced by amniotes such as parareptiles or eureptiles (Fig. 1e, f, k; Fig. S11, S12). Other footprints cannot be confidently attributed to Ichno-genus due to poor preservation, but they represent a variety of morphology and sizes (5–17 cm) (Fig. 1j, h, l, m; Fig. S13–S16).

**Figure 1.**
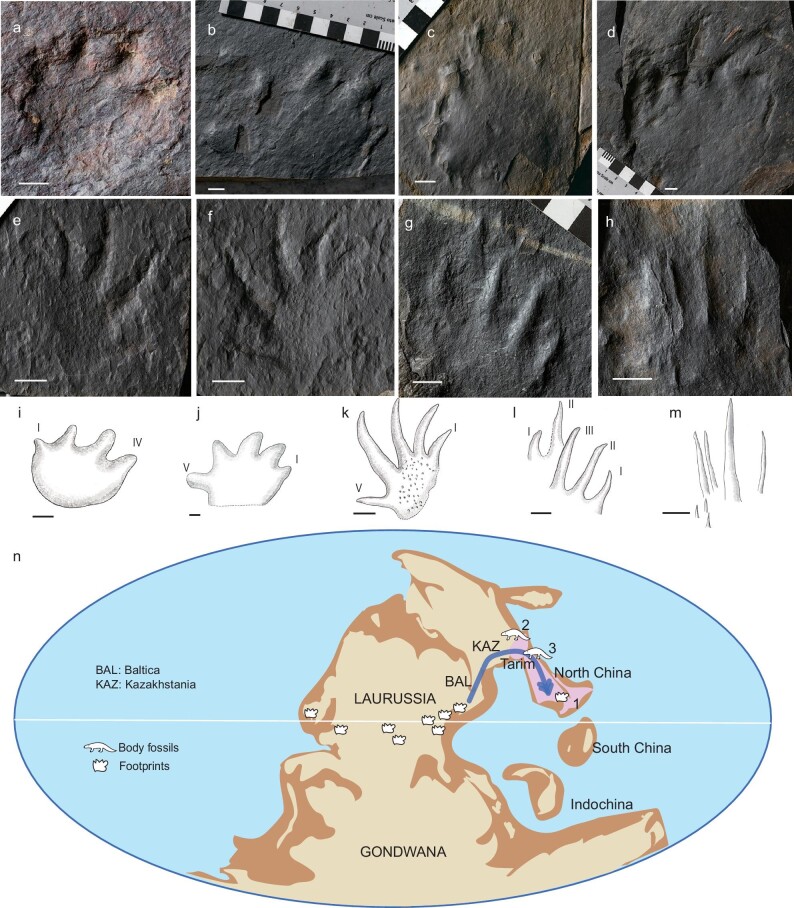
Tetrapod imprints from the early Permian Mentougou Ichno-fauna of North China. (a)–(h) Photos of imprints: (a)–(d), *Limnopus* (IVPP V31904, 31905–1, 31906); (e) and (f), amniote imprint (IVPP V31907); (g) and (h), unidentified imprint (IVPP V31908, 31909). (i)–(m) Line drawings of imprints: (i), medium-sized *Limnopus* manus (IVPP V31904); (j), large *Limnopus* pes (IVPP V31905-1); (k), amniote imprint (IVPP V31907); (l), unidentified imprint (IVPP V31908); (m), unidentified imprint (IVPP V31909). Scale bars equal 1 cm. (n) Reconstructed global paleogeography of early Permian, modified from various sources [10,12]. The pink region highlights the Tarim Block and North China Block. The numbers represent: 1. Mentougou Ichno-fauna (298 Ma); 2. *Urumqia liudaowanensis* (285 Ma); 3. Dashankou fauna (268 Ma). The footprint symbols mark the global occurrences of *Dromopus* biochron, including the new discovery of Mentougou Ichno-fauna [11]. The blue line with arrow represents the proposed dispersal route through a higher latitude.

The footprint combination probably belongs to the *Dromopus* biochron, which was widely distributed in central and western Pangea during the late Carboniferous–early Permian (Fig. 1n) [11]. For the whole fauna to be also distributed in the far east of Pangea, a free land movement must have existed between NCB and the rest of Pangea by 298 Ma. In other words, it provides strong evidence that NCB was connected with the Tarim Block prior to early Permian, supporting the ‘Early Collision’ hypothesis [2,3].

A dispersal route from tropical central Pangea through a higher latitude Baltica, Kazakhstania and Tarim Blocks to the NCB was proposed (Fig. 1n, Fig. S17). Some other evidence supports this route. A seymouramorph *Urumqia liudaowanensis* occurred in the Lucaogou Formation (>285 Ma) of southern Jungar Basin (Fig. 1n, Fig. S17). It was the oldest body fossil of tetrapods from northern China, and constrained the collision between Baltica and Kazakhstania to be earlier than 285 Ma [6]. A single footprint from Baode, Shanxi, probably belonging to *Dimetropus*, was originally reported from the Carboniferous but later revised to be from the Lower Shihhotse Formation of early Permian [6] (Fig. S17). The age was recently dated as ∼295 Ma [8]. Diatectomorphs and embolomeres were two tetrapod clades that mostly flourished in the late Carboniferous–early Permian tropical Central Pangean Mountains. Curiously, however, out of their ‘normal’ temporal and geographic distribution, a diatectomorph (*Alveusdectes fenestratus*) and an embolomere (*Seroherpeton yangquanensis*) were discovered from the late Permian (∼252 Ma) of Jiyuan, Henan Province and Yangquan, Shanxi Province of NCB, respectively [12,13] (Fig. S17). With the discovery of tetrapod footprints in the early Permian Mentougou Ichno-fauna, it showed that tetrapods already existed in NCB by 298 Ma, a time in accordance with their ‘normal’ occurrences in Pangea. In a word, the new discovery supports a Pangean cosmopolitanism, rather than provincialism, for the evolution of tetrapods from as early as the Asselian. We predict further body fossil discoveries from the terrestrial sediments of early Permian or even Carboniferous of east Pangea.

Among the recognized footprint types in the new Ichno-fauna, IVPP V31907 is produced by amniotes such as parareptiles or eureptiles (Fig. 1e, f, k). They likely represent the earliest record of amniotes from east Pangea, predating the earliest body fossil of amniotes from the Dashankou fauna of Gansu, China by 30 million years (Fig. 1n, Fig. S17) [6]. Amniotes are highly adapted to terrestrial lifestyles, and their origin and early radiation has long been associated with the formation of the supercontinent Pangea [14]. They first occurred at west Pangea at ∼318 Ma. By the start of Permian, they were diversified into synapsids and sauropsids and were widely distributed in central and west Pangea [14]. However, no amniote fossil was previously known from east Pangea during the late Carboniferous and early Permian [6]. The probable amniotes in Mentougou Ichno-fauna of early Permian differs from those of Dashankou fauna, in which the amniotes were more diversified and represented by dinocephalians, anomodonts, captorhinids and bolosaurids [6]. The difference in the two fauna documents at least one previously unknown faunal turnover between early-middle Permian (298–268 Ma) in east Pangea, the Olson's Extinction [15].

With strong evidence supporting a connected NCB-Pangea in early Permian, its implication for paleoclimate should be considered. The Paleo-Asian Ocean was integrated when NCB and Tarim Block remained separate, but once the two became connected and subsequently collided with Pangea, the south-north ocean flow of the Paleo-Asian Ocean was closed. The Pangean megamonsoon started in the late Carboniferous and reached its peak in the Triassic. It was considered a major factor in the aridification process, the disappearance of the coal, the warming, and the increased seasonality during the Permian [7], like the effect of the present-day East Asian Monsoon but to a different degree [9]. A recent study shows that land configuration had been a main factor in the intensity of the megamonsoon system in Pangea [9]. A united NCB-Pangea may imply a more intense monsoon system at east Pangea during the early Permian, than the result based on a fragmented NCB-Pangea model. A recent study found that the floral history between NCB and Euramerica was similar in the early Permian, with the disappearance of coals and tropical flora occurring much earlier than previously thought in the Cisuralian [8]. By comparing the composition of Mentougou Ichno-fauna and Dashankou fauna, responses of terrestrial tetrapods seem to couple those of plants. The Mentougou Ichno-fauna was found in the uppermost Taiyuan Formation, together with coal seams and typical Cathaysian plants; the coals disappeared at the overlying Shansi Formation. The climate at 298 Ma in NCB was warm and humid; by 268 Ma, it had become seasonally hot and more arid [8]. Whether and how a united NCB-Pangea affects paleoclimate during early Permian by increasing the intensity of the megamonsoon, reducing the coastline, shifting the landscape of the subduction zone, and closing the ocean flow warrants further scrutiny.

## Supplementary Material

nwae249_Supplemental_File

## References

[1] Crowley TJ . In: Klein GO (ed.). Pangea: Paleoclimate, Tectonics, and Sedimentation during Accretion, Zenith, and Breakup of a Supercontinent Vol. 288. New Jersey: Geological Society of America, 1994. 25–56.

[2] Domeier M, Torsvik TH. Geosci Front 2014; 5: 303–50. 10.1016/j.gsf.2014.01.002

[3] Wilhem C, Windley BF, Stampfli GM. Earth-Sci Rev 2012; 113: 303–41. 10.1016/j.earscirev.2012.04.001

[4] Scotese CR . in PALEOMAP Atlas for ArcGIS. PALEOMAP Project. 2014. www.scotese.com

[5] Xiao W, Windley BF, Sun S et al. Annu Rev Earth Planet Sci 2015; 43: 477–507. 10.1146/annurev-earth-060614-105254

[6] Liu J, Yi J, Chen J-Y. Earth-Sci Rev 2020; 207: 103215. 10.1016/j.earscirev.2020.103215

[7] Tabor NJ, Poulsen CJ. Palaeogeogr Palaeoclimatol Palaeoecol 2008; 268: 293–310. 10.1016/j.palaeo.2008.03.052

[8] Wu Q, Ramezani J, Zhang H et al. Geology 2021; 49: 677–81. 10.1130/G48051.1

[9] Hu Y, Li X, Boos WR et al. Nat Geosci 2023; 16: 1041–6. 10.1038/s41561-023-01288-y

[10] Colbert EH In: Turner MD, Splettstoesser JF (eds). Geology of the Central Transantarctic Mountains. Washington DC: American Geophysical Union, 1986. 11–35.

[11] Voigt S, Lucas SG. Geol Soc Spec Publ 2018; 450: 387–404. 10.1144/SP450.10

[12] Liu J, Bever GS. Biol Lett 2015; 11: 20150100. 10.1098/rsbl.2015.0100 25948572 PMC4455737

[13] Chen J, Liu J. Foss Rec 2020; 23: 205–13.

[14] Carroll RL . Vertebrate Paleontology and Evolution. New York: WH Freeman, 1988.

[15] Brocklehurst N . Proc R Soc Lond B Biol Sci 2020; 287: 20200154. 10.1098/rspb.2020.0154 PMC7341920 32517621

