# Supplementary material for: Journey to the east: the oldest tetrapod fauna of east Pangea in early Permian
Source: Natl Sci Rev. 2024 Jul 23;11(10):nwae249. doi: 10.1093/nsr/nwae249 (PMC11441314; doi:10.1093/nsr/nwae249)
Supplement: nwae249_Supplemental_File [file nwae249_supplemental_file.docx]

**Supplementary Data: Material and Methods**

The fossil footprints were collected from the dumping slabs during the road construction of G109 since 2021. This site lies in the suburb of Beijing (N 39°59′24.18″, E 116°4′31.78″), ~30 km west to the Forbidden City.

The fossil footprints were preserved on dark grey siltstone with horizontal bedding, together with abundant fossil plants such as *Lepidodendron*, *Sphenophyllum*, *Annularia*, *Pecopteris*, and *Neuropteris*. These plants were mostly Pennsylvanian in age but could also extend to Asselian [1]. The siltstone includes small iron-oxide nodules. The combination of fossil plants and iron-oxide nodules correlates the fossil-bearing layer to bed 26 of the so-called Huiyu section [2], equal to the uppermost bed of the Taiyuan Formation [1]. The top of the Taiyuan Formation is recently dated as 298.18± 0.32 Ma at Baode, Shanxi [3], and this layer at Beijing is roughly isochronus [1, 3], so the footprints is the earliest Asselian in age (~298 Ma). A summary of the ages and localities of fossil tetrapods in Northern China can be found in Suppl. Fig. 17.

A total of six slabs were cataloged (IVPP V31904-31909; Suppl. Fig. 1-16), with each preserving one or more footprints with variable qualities. Although most footprints could not be confidently identified, some better-preserved footprints can be attributed to two ichnogenera, *Limnopus* and cf. *Tambachichinium*. Below is a description of some representative footprints.

*Limnopus* Marsh, 1894 [4] (Fig. 1a-d; Suppl. Fig. 1-8)

**Referred specimens**. IVPP V31904-31906

**Description and Discussion**.

Two imprints are confidently identified as *Limnopus*, and many less well-preserved ones may also belong to *Limnnopus*. They are characterized by plantigrade imprints with short and broad digits, distally rounded and departing radially from palm. Sizes vary from 5 to 15 cm.

Among the two better preserved footprints, the smaller one (IVPP V31904, Fig. 1a, i; Suppl. Fig. 2, 3) is a manus with four digits, with sub-equal length and width of 4.5 cm. The length of the digits are as follows: III>IV=II>I.

The larger one (IVPP V31905-1, Fig. 1b, j; Suppl. Fig. 4, 5) is one of the three imprints preserved on IVPP V31905 (Suppl. Fig. 1). It is about 10 cm wide, preserved in convex hyporelief and concave epirelief. It represents a pes as evidenced by five digits, with the sole well impressed but only partially preserved. Digit imprints I-III are closely grouped. Digital lengths increase from I to IV, and digit V is subequal to digit II. Digit imprint I is the thinnest whereas digit V is the thickest. Digits I to III slightly turn inward, and digit V lies posteriorly to the other digits.

IVPP V31905-2 and IVPP V31905-3 are two imprints that may also belong to *Limnopus*, although they are less well preserved than IVPP V31905-1. V31905-2 (Fig. 1d; Suppl. Fig. 6, 7) is an isolated plantigrade pentadactyl imprint with the length and width of 15x12 cm, preserved in convex hyporelief and concave epirelief. Digits have tapered tips, and increase in length from I to IV. Digit V is similar in length with digit II but much stouter. All five digits are slightly turned outward. IVPP V31905-3 (Suppl. Fig. 8, 9) is a large incomplete imprint. It is slightly wider than long, and the width is ~14 cm. Only four rounded dight tips are faintly preserved, suggesting that it is a manus.

IVPP V31906 (Fig. 1c; Suppl. Fig. 10) are two medium-sized plantigrade imprints superimposed, A total of seven short digits can be counted. Digit impressions are wide and straight with rounded tips, but the tips are not expanded. Based on the short digits with rounded tips, the two imprints are most likely *Limnopus*, although the possibility of *Amphisauropus* and *Ichniotherium* cannot be completely excluded.

The potential track makers for *Limnopus* are large anamniotes, especially temnospondyls such as eryopids [5-7].

Definite amniote imprint (Fig. 1e, f, k; Suppl. Fig. 11, 12)

**Referred specimens**. IVPP V31907

**Description and Discussion**.

It is a single medium sized (~5 cm) plantigrade pentadactyl imprint preserved in convex hyporelief and concave epirelief. It is longer than wide. Digits are long and slender with tapered tips. Length increases from digit I to IV, and digit V is as long as I. Digits I to IV are equally spaced and curved slightly inward; digit V is straight and widely spaced from digit IV. Scale impressions are clearly visible from the palm/sole.

This type of imprints represents amniote tracks [5, 7, 8, 9], likely produced by parareptiles or eureptiles. It resembles *Tambachichnium*, *Notalacerta*, or *Varanopus*, but without pairs or trackways, it is difficult to assign it to the exact ichno-genus.

Unidentified tracks

Besides the imprints mentioned above, many imprints cannot be attributed to any ichno-genus due to lack of identifiable features.

IVPP V31904 (Suppl. Fig. 2) preserves over 10 footprints and one possible tail traces, but other than one imprint attributed to *Limnopus*, all the others cannot be confidently assigned to any ichno-genus. They are medium to large imprints, some of which form possible manus/pes pairs.

IVPP V31908 (Fig. 1g, Suppl. Fig. 13, 14) are represented by two incomplete small plantigrade imprints in convex hyporelief and concave epirelief. A total of five digits are visible. We interpret that the left two digits belong to one hand/foot, and the right three digits with a palm/sole belong to the other, based on their relative lengths and their curving direction. Digit I curves laterally whereas digits II and III curves medially. The length increases from I to III.

IVPP V31909 (Suppl. Fig. 15) preserves a few small (<5 cm), straight and slender digits parallel to each other, and some pointed digit tips. Another uncatalogued block preserves one medium-sized imprint with some digits with rounded tips and a palm/sole (Suppl. Fig. 16), but it cannot be determined whether it represents *Amphisauropus*, *Limnopus* or *Ichniotherium*.

**Reference**

1. Shen B, Shen S, Wu Q *et al.* Carboniferous and Permian integrative stratigraphy and timescale of North China Block. *Science China Earth Sciences*. 2022; **65**(6): 983-1011.

2. Bureau of Geology and Mineral Resources of Beijing Municipality, Geological Publishing House, Beijing, 1991: 1-598.

3. Wu Q, Zhang H, Ramezanni J *et al.* High-precision U-Pb age constraints on the Permian floral turnovers, paleoclimate change, and tectonics of the North China block. *Geology.* 2021; **49**: 677-681. doi:10.1130/g48051.1

4. Marsh OCI. Footprints of vertebrates in the coal-measures of Kansas. *Geological Magazine.* 1894; **1**: 337-339.

5. Voigt S, Lucas SG. Outline of a Permian tetrapod footprint ichnostratigraphy. *Geological Society, London, Special Publications.* 2018; **450**: 387-404. doi:10.1144/sp450.10.

6. Voigt S, Haubold H. Permian tetrapod footprints from the Spanish Pyrenees. *Palaeogeography, Palaeoclimatology, Palaeoecology.* 2015; **417**: 112-120.

7. Voigt S, Lucas SG. Permian tetrapod ichnodiversity of the Prehistoric Trackways National Monument (south-central New Mexico, USA). *New Mexico Museum of Natural History and Science Bulletin.* 2015; **65**: 153-167.

8. Hermann Müller A. Zur Ichnologie und Stratonomie des Oberrotliegenden von Tambach (Thüringen). *Paläontologische Zeitschrift.* 1954; **28**: 189-203.

9. Voigt S. *Die tetrapodenichnofauna des kontinentalen oberkarbon und perm im thüringer Wald-ichnotaxonomie, paläoökologie und biostratigraphie*: Cuvillier Verlag, 2005.


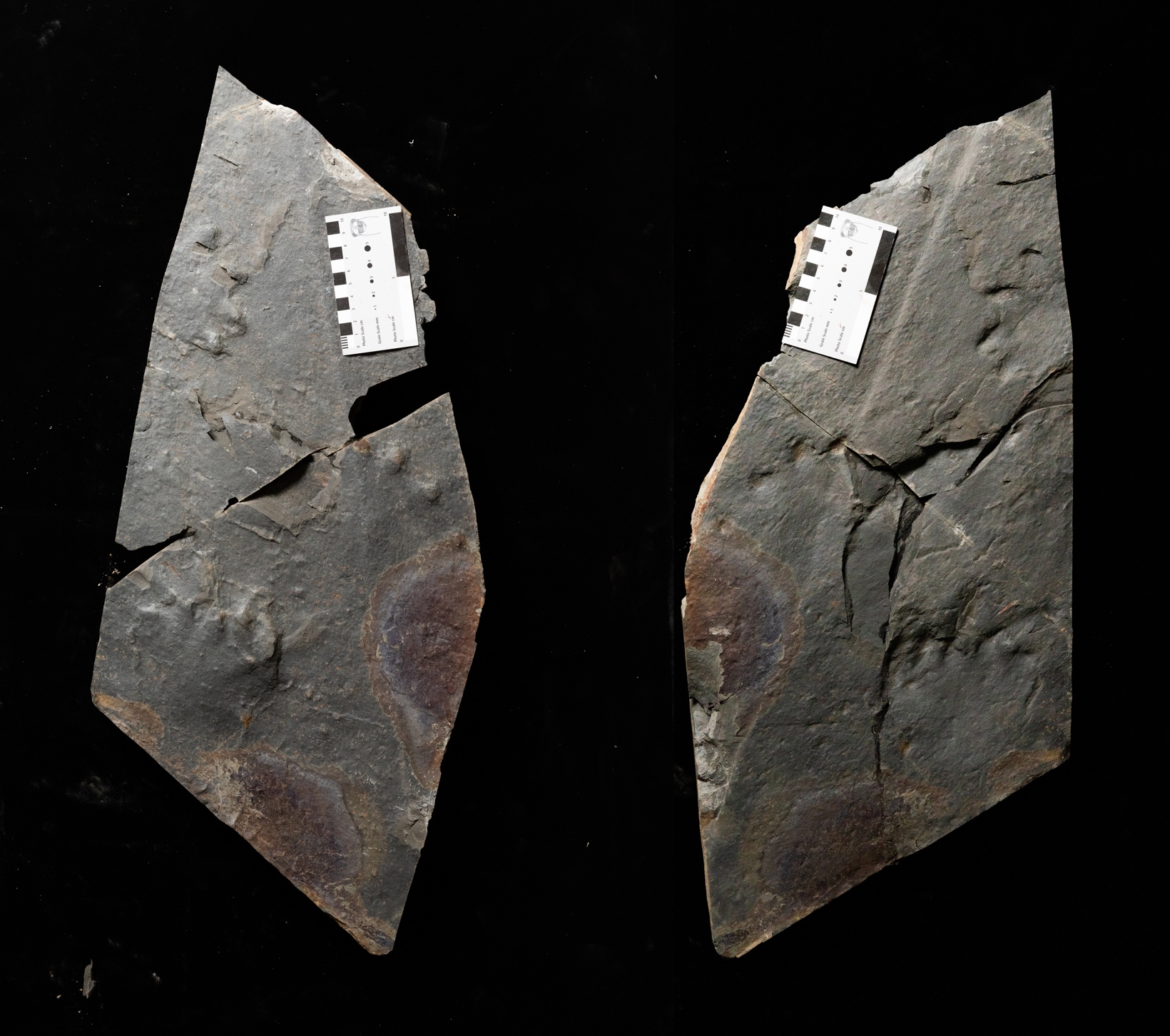


Supplementary Figure 1. IVPP V31905, part and counterpart, showing three footprints of *Limnopus*.


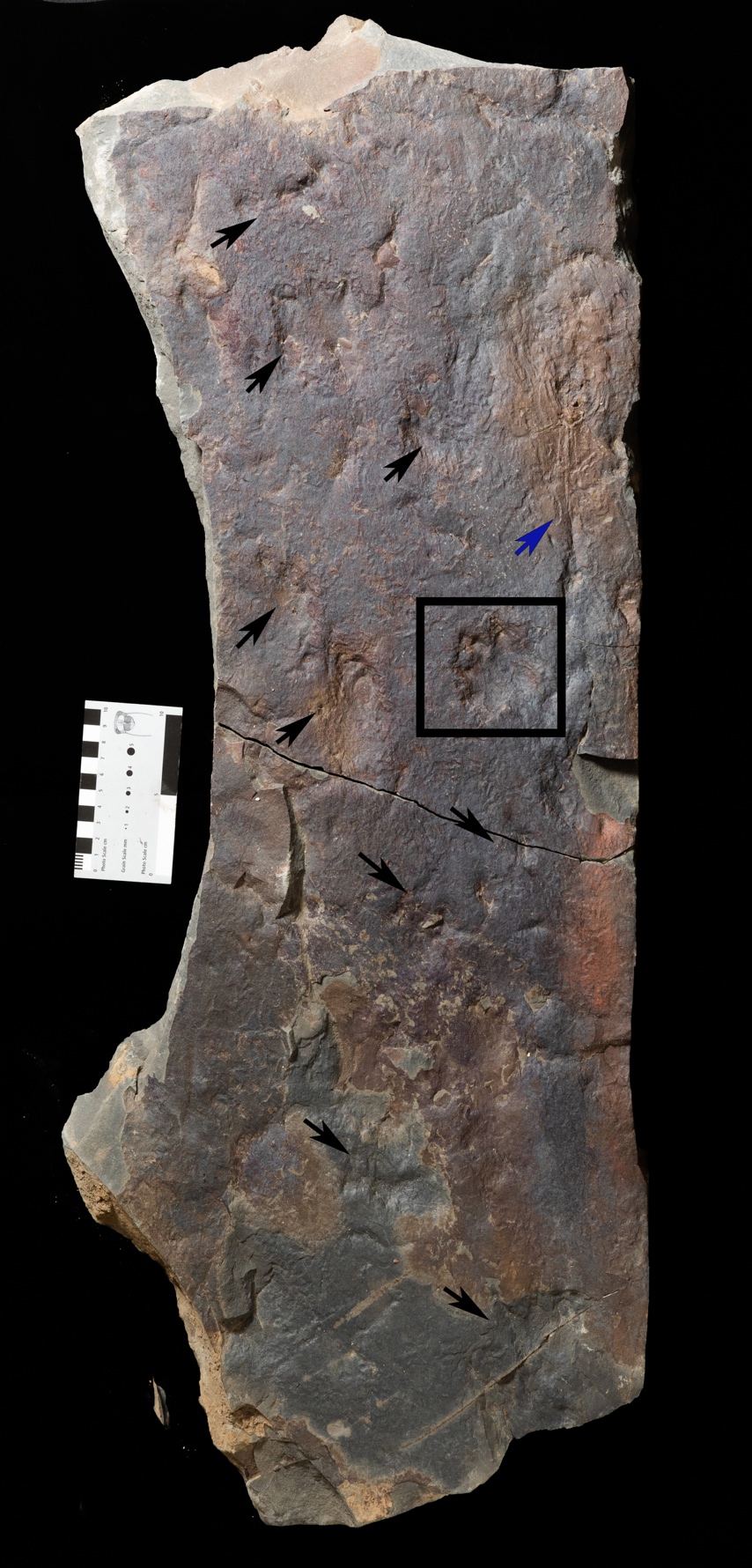


Supplementary Figure 2. c, IVPP V31904, showing footprints of *Limnopus* (in black box), some unidentifiable footprints (black arrows), and a possible tail trace (blue arrow).

Supplementary Figure
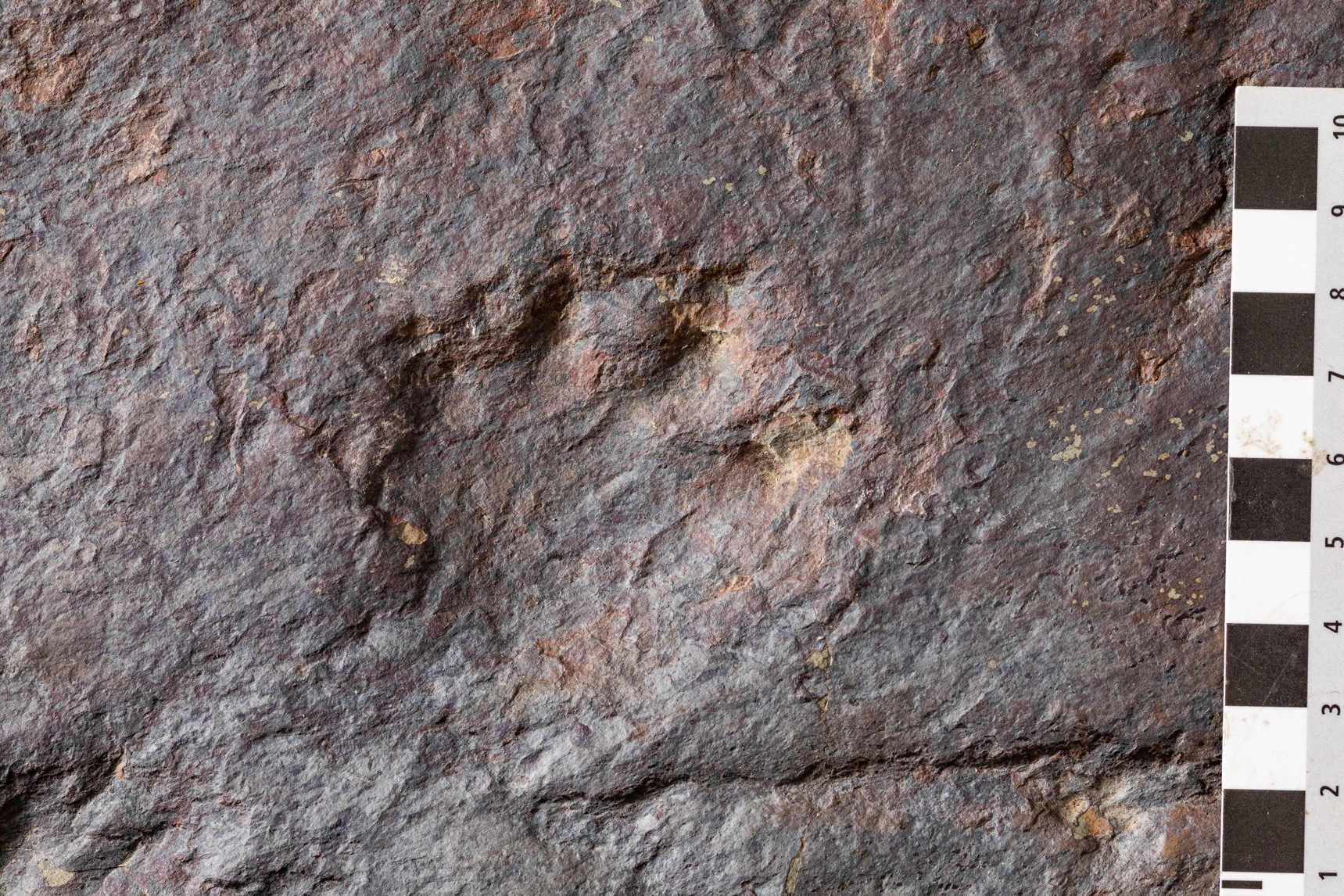
3. IVPP V31904, *Limnopus* manus imprint.

Supplementary Figure
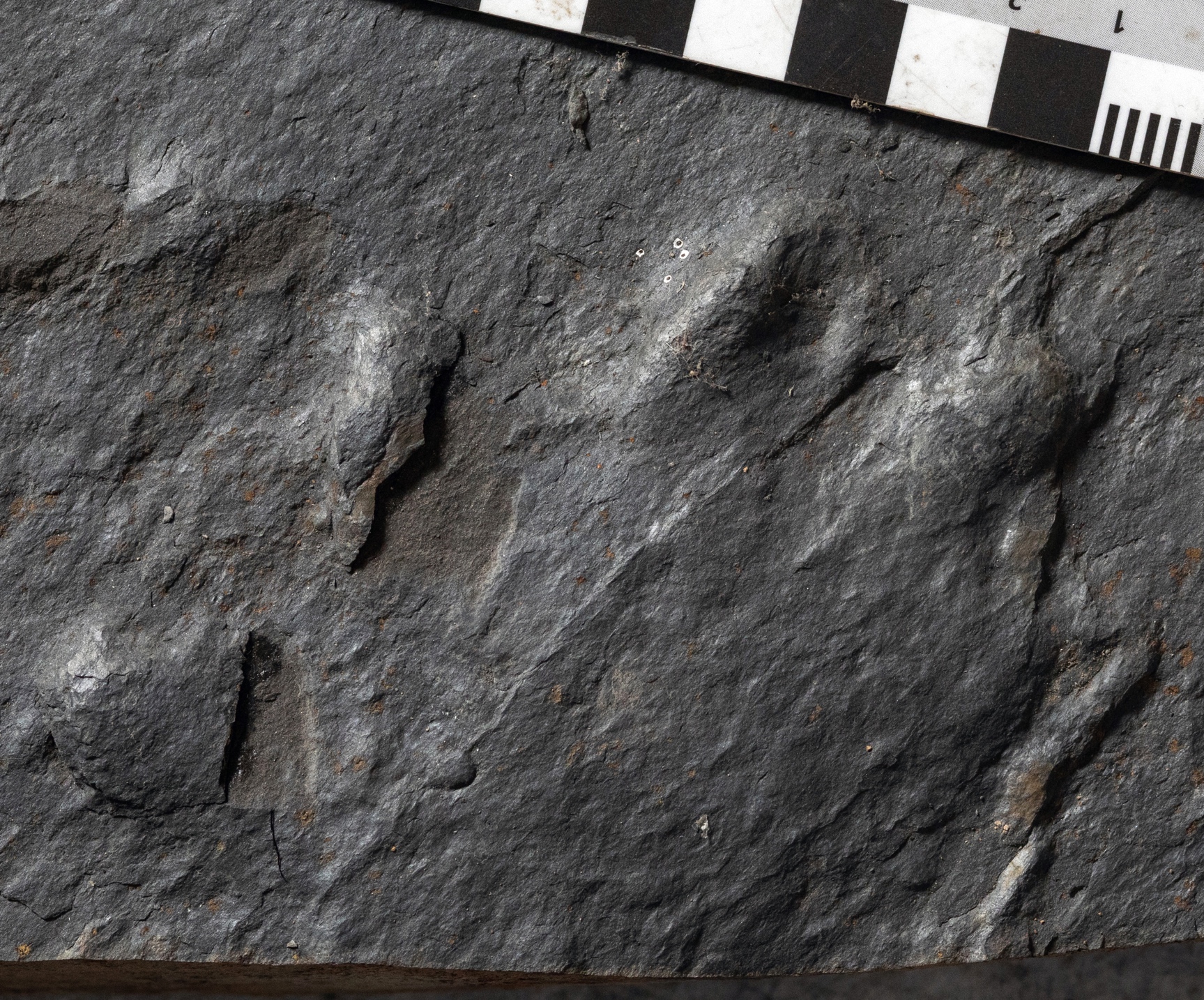
4. IVPP V31905-1a, *Limnopus* pes imprint in convex hyporelief.


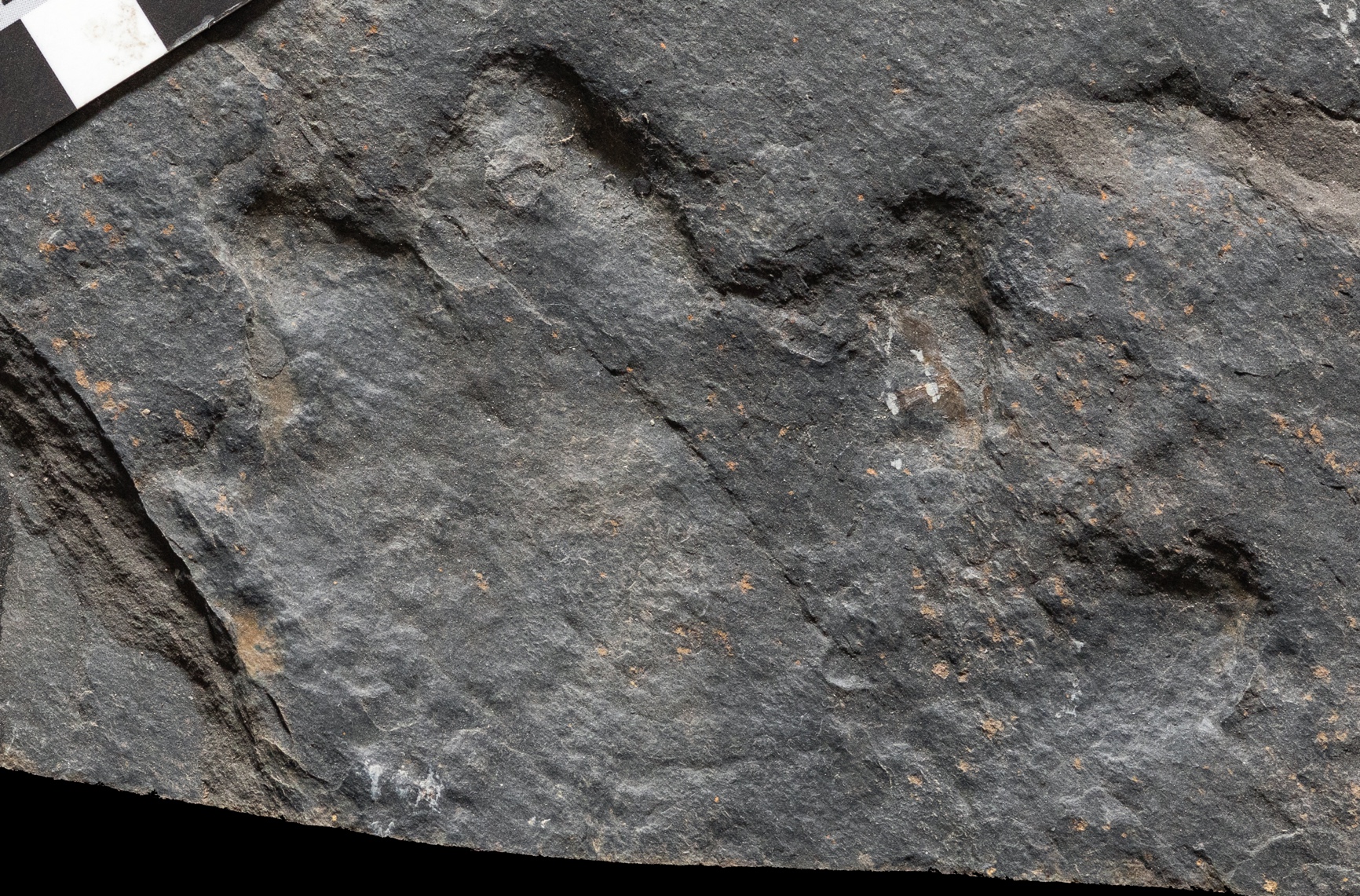


Supplementary Figure 5. IVPP V31905-1b, *Limnopus* pes imprint in concave epirelief.


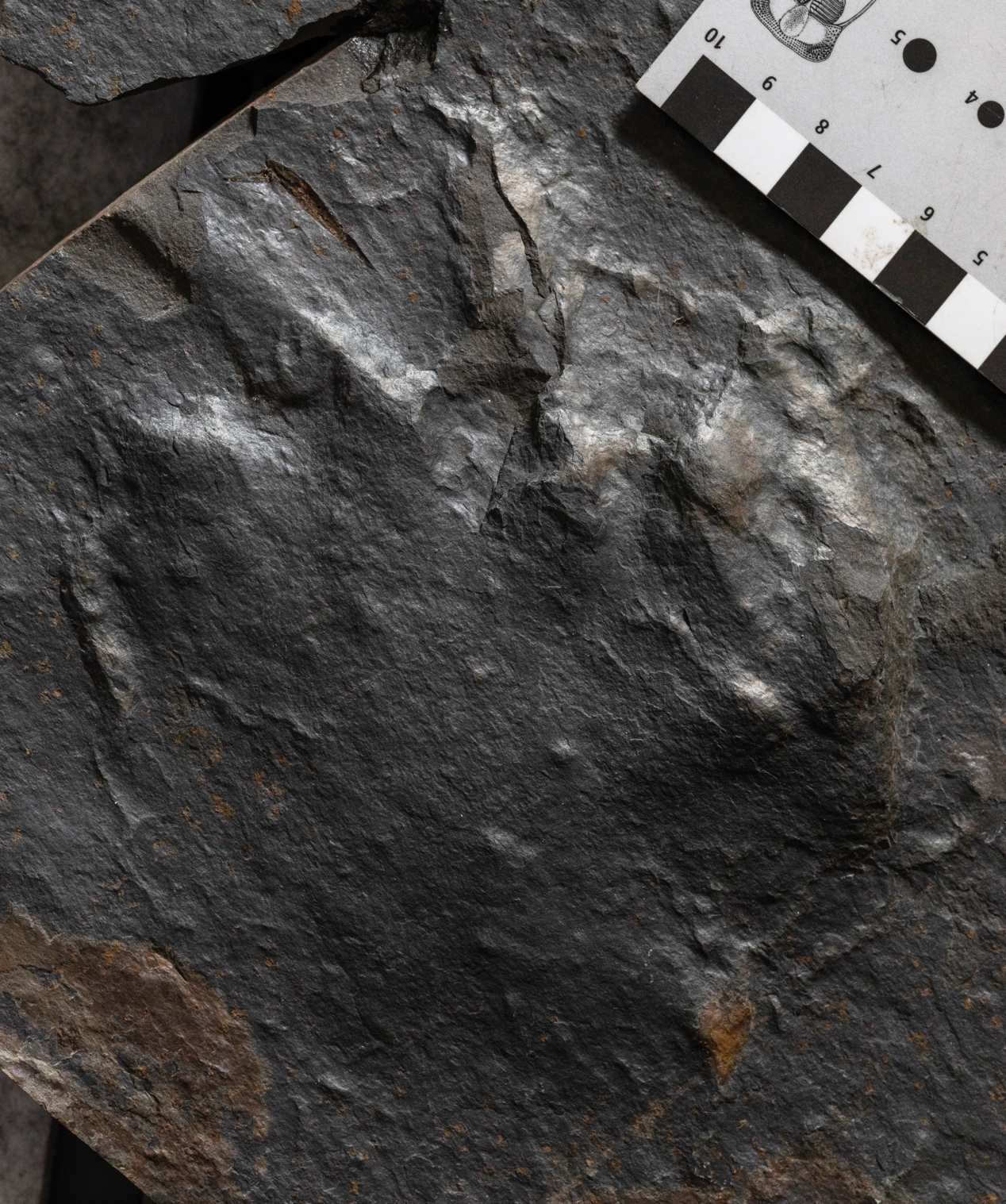
Supplementary Figure 6. IVPP V31905-2a, a possible *Limnopus* pes imprint in convex hyporelief.


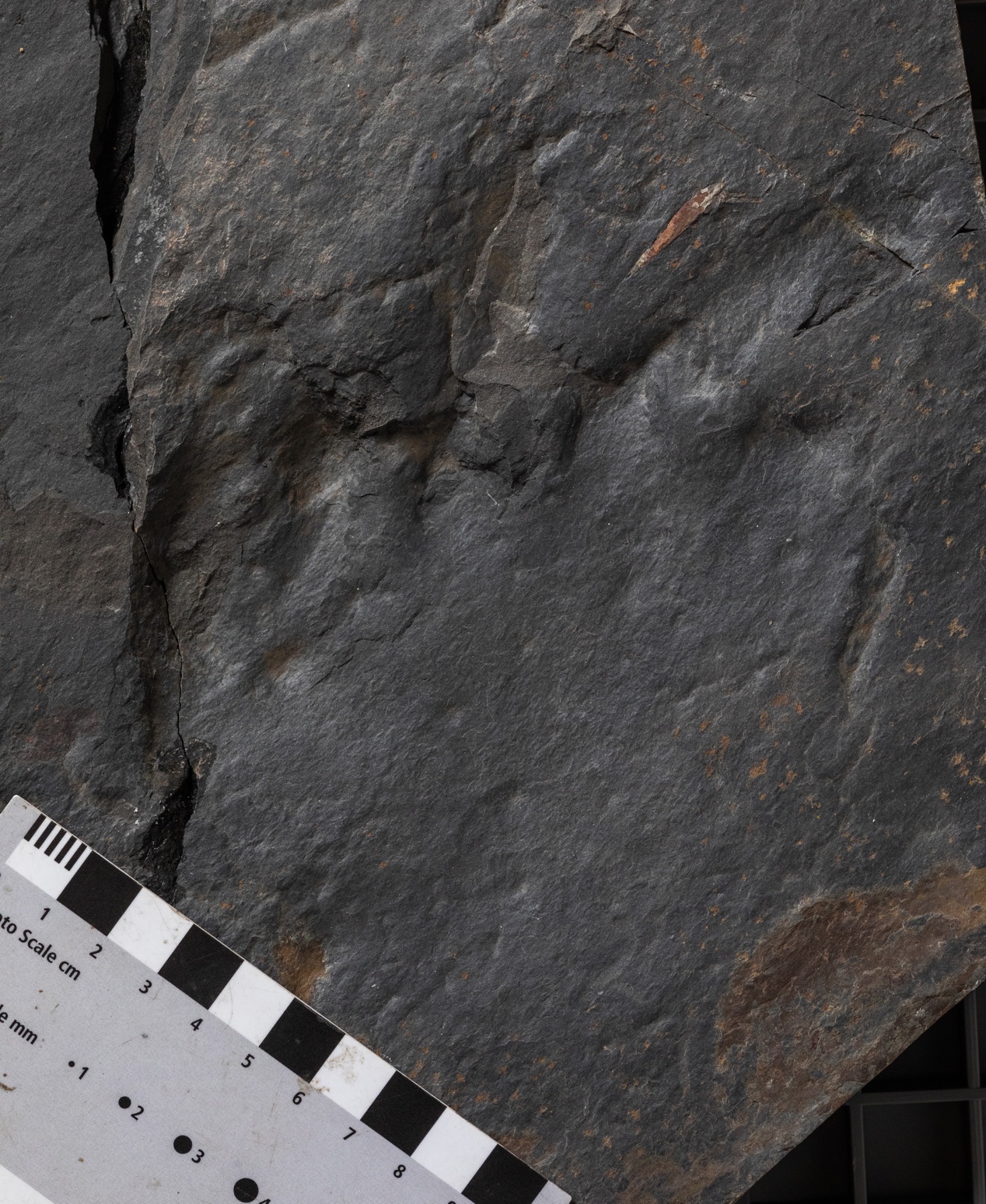


Supplementary Figure 7. IVPP V31905-2b, a possible *Limnopus* pes imprint in concave epirelief.


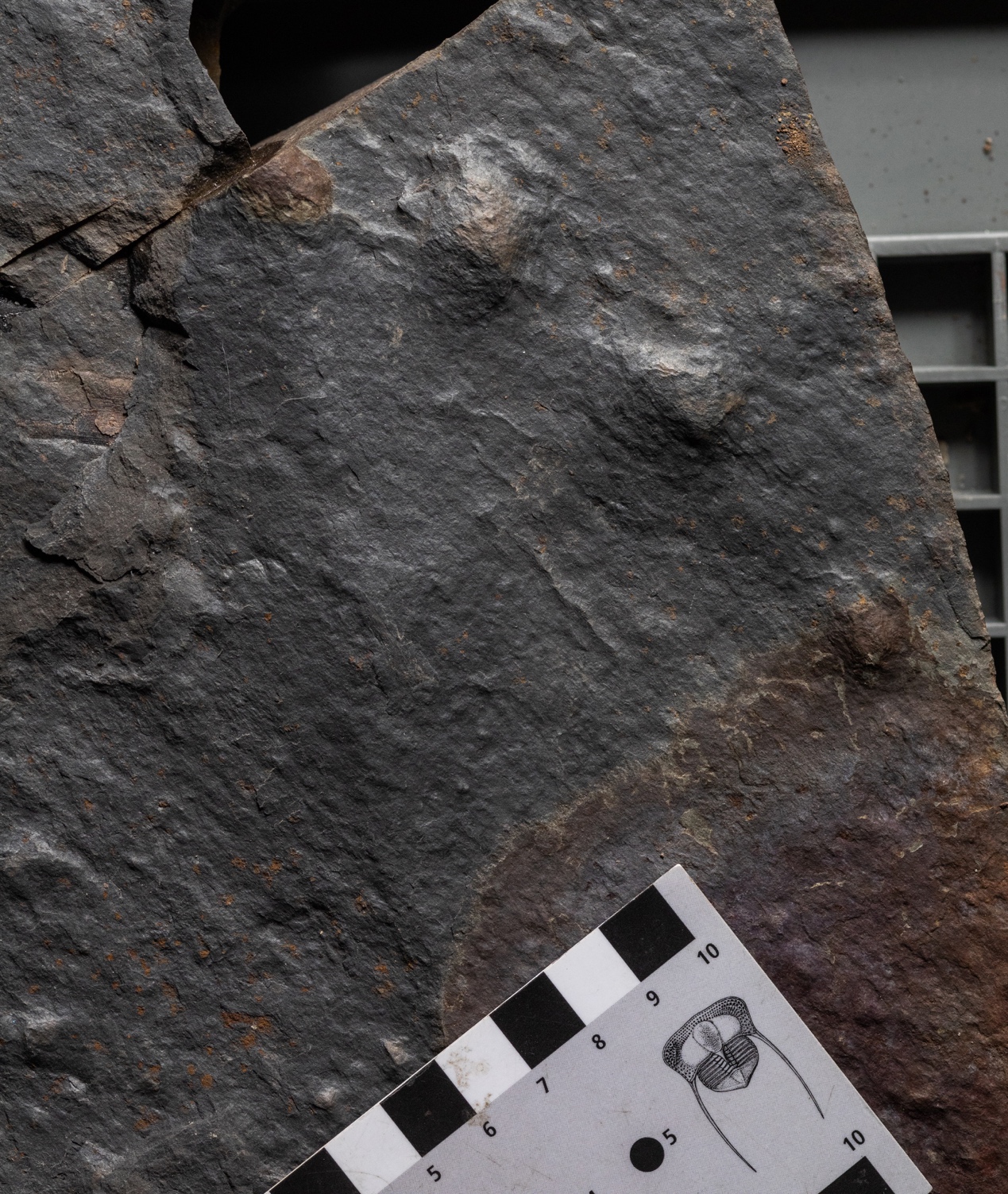


Supplementary Figure 8. IVPP V31905-3a, a possible *Limnopus* manus imprint in convex hyporelief.


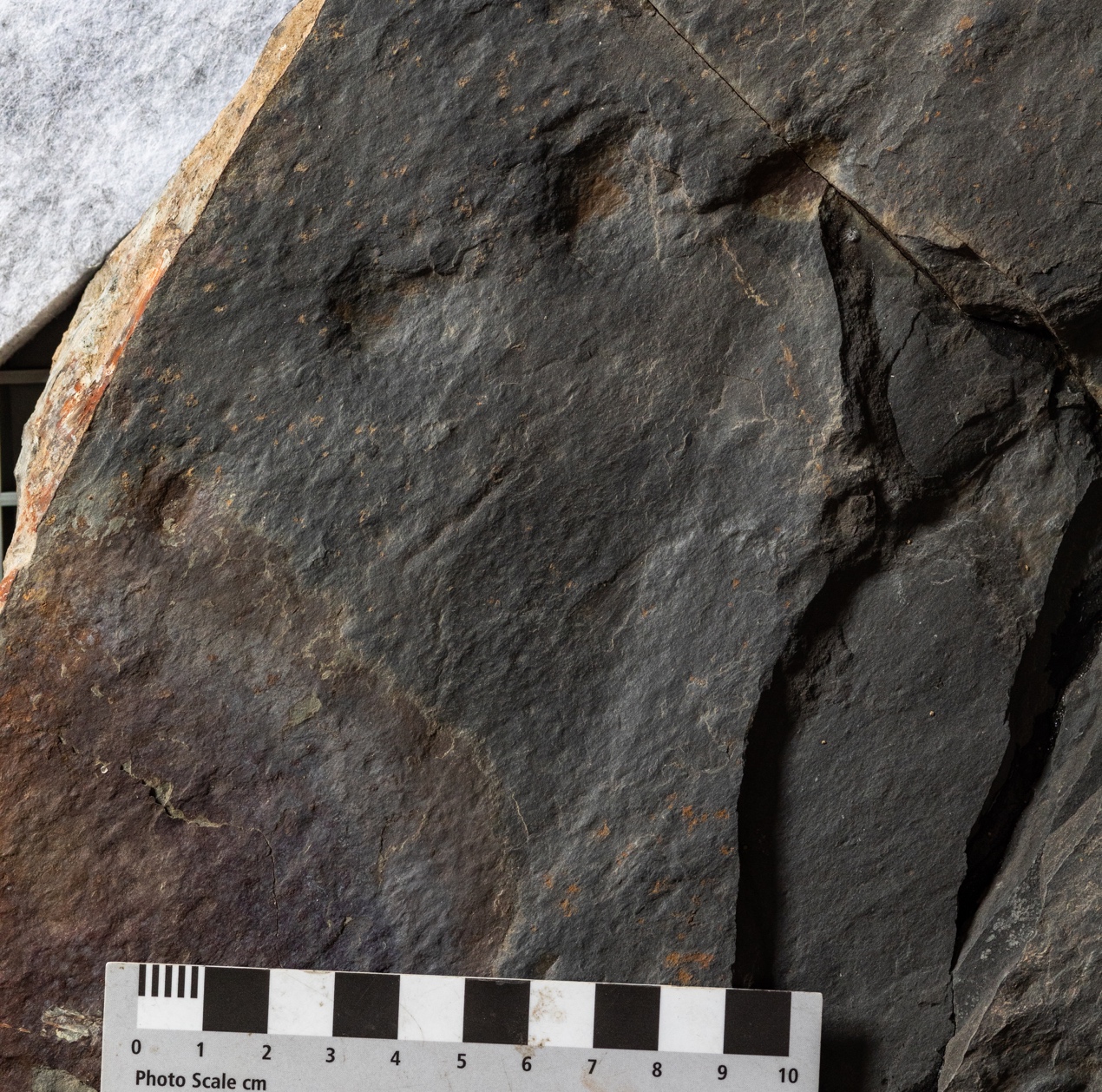


Supplementary Figure 9. IVPP V31905-3b, a possible *Limnopus* manus imprint in concave epirelief.


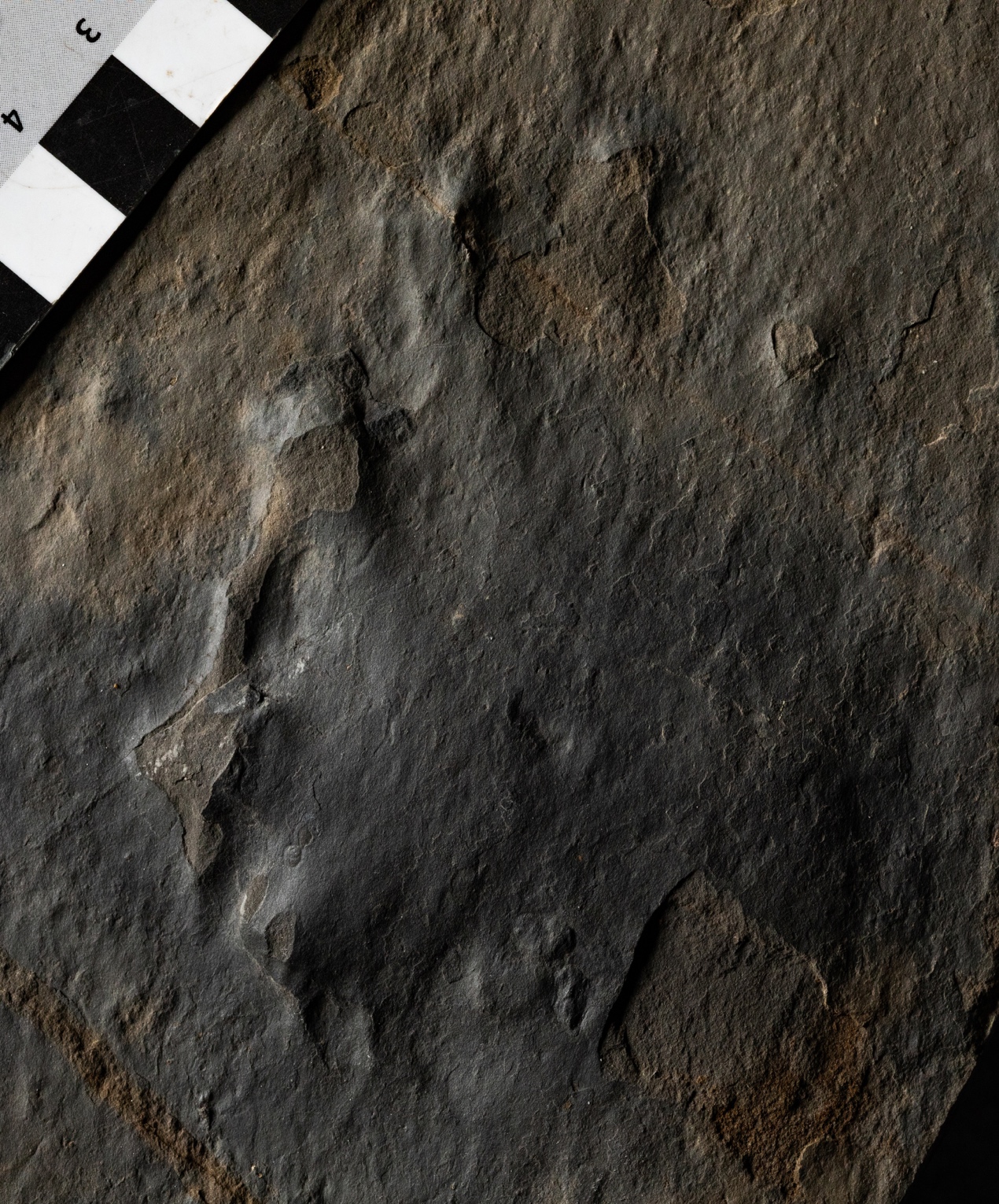


Supplementary Figure 10. IVPP V31906, two partial imprints superimposed, possibly belonging to *Limnopus*.


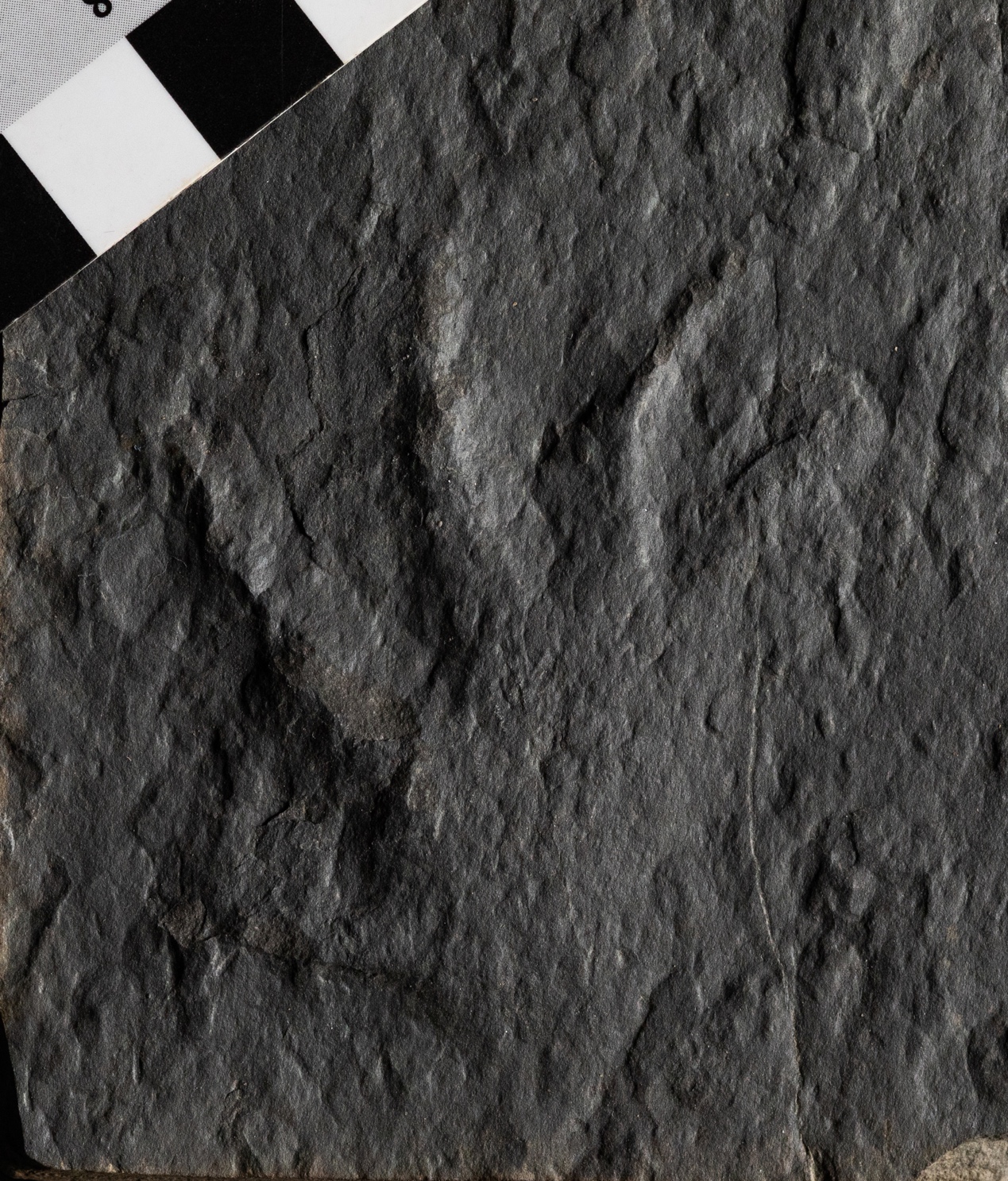


Supplementary Figure 11. IVPP V31907a, an amniote imprint in convex hyporelief.


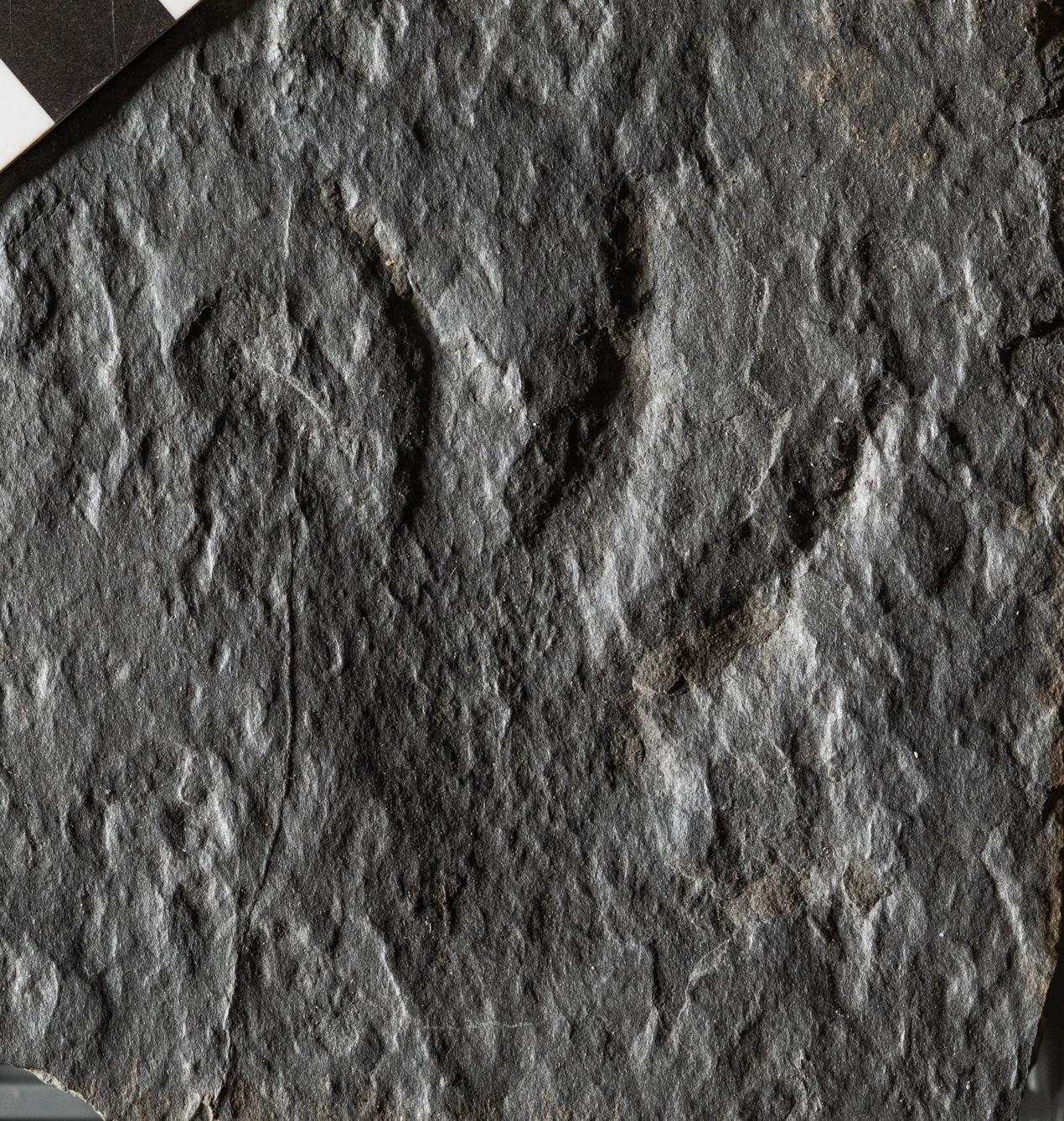


Supplementary Figure 12. IVPP V31907b, an amniote imprint in concave epirelief.


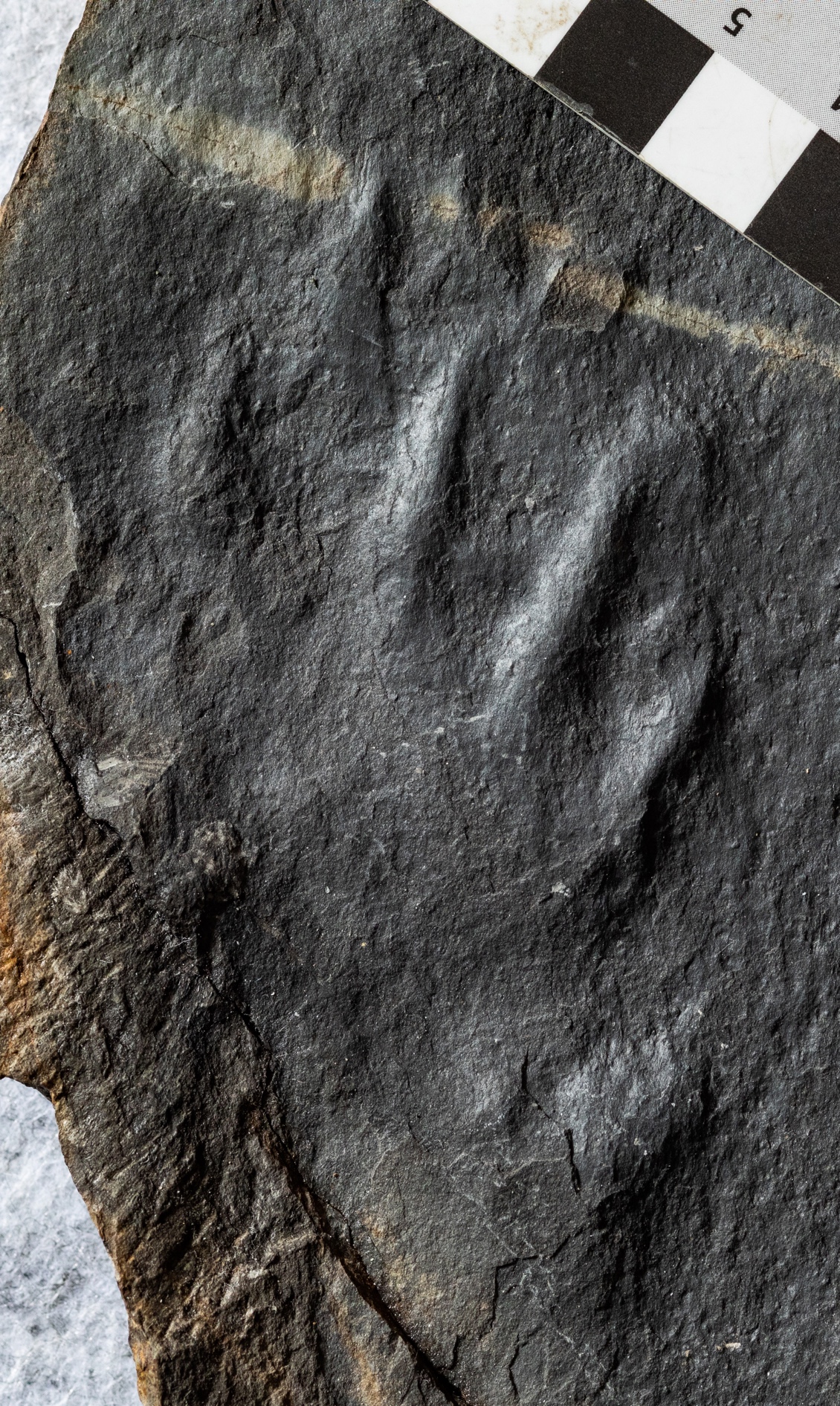


Supplementary Figure 13. IVPP V31908a, an unidentified imprint in convex hyporelief.


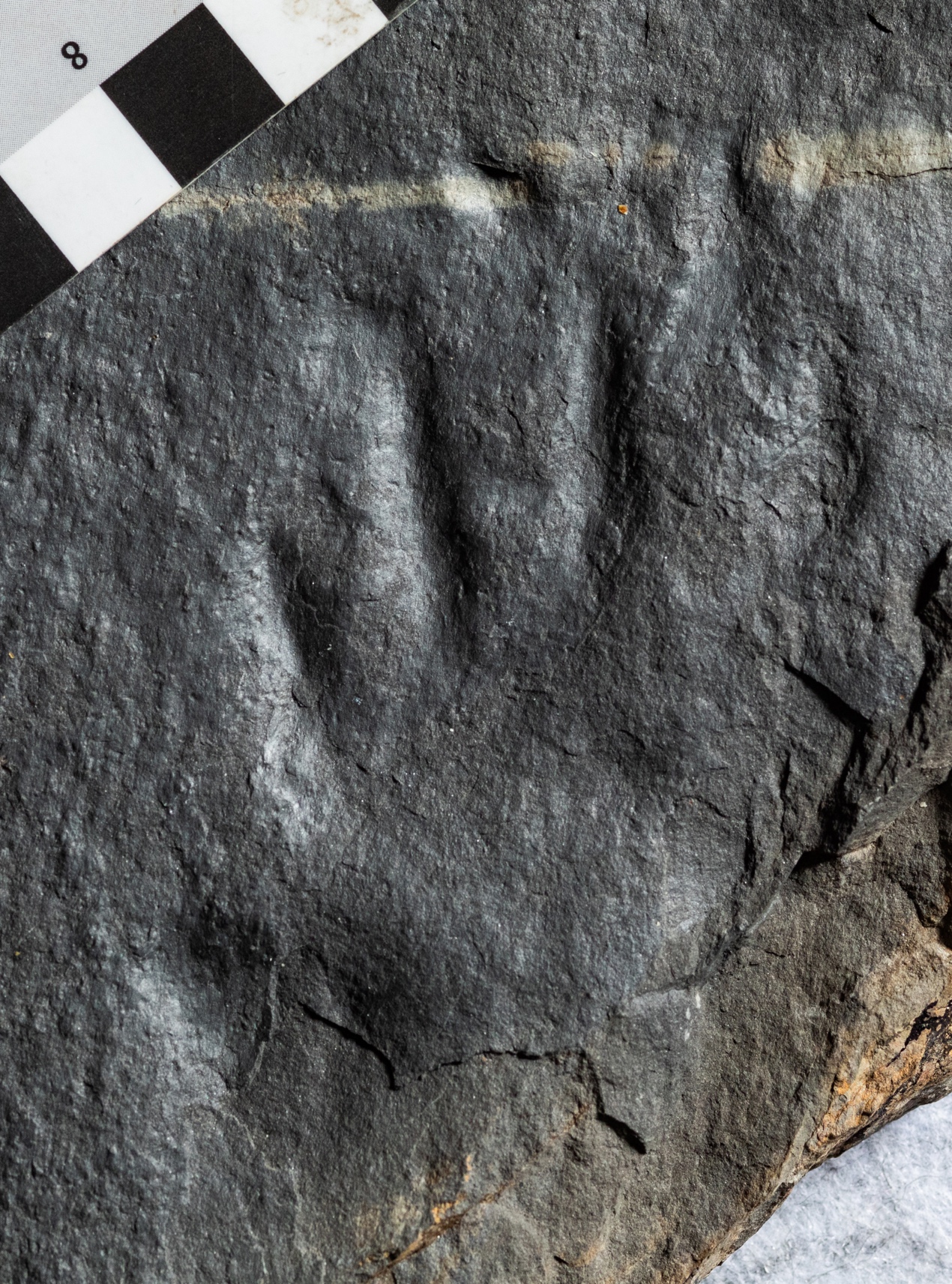


Supplementary Figure 14. IVPP V31908b, an unidentified imprint in concave epirelief.


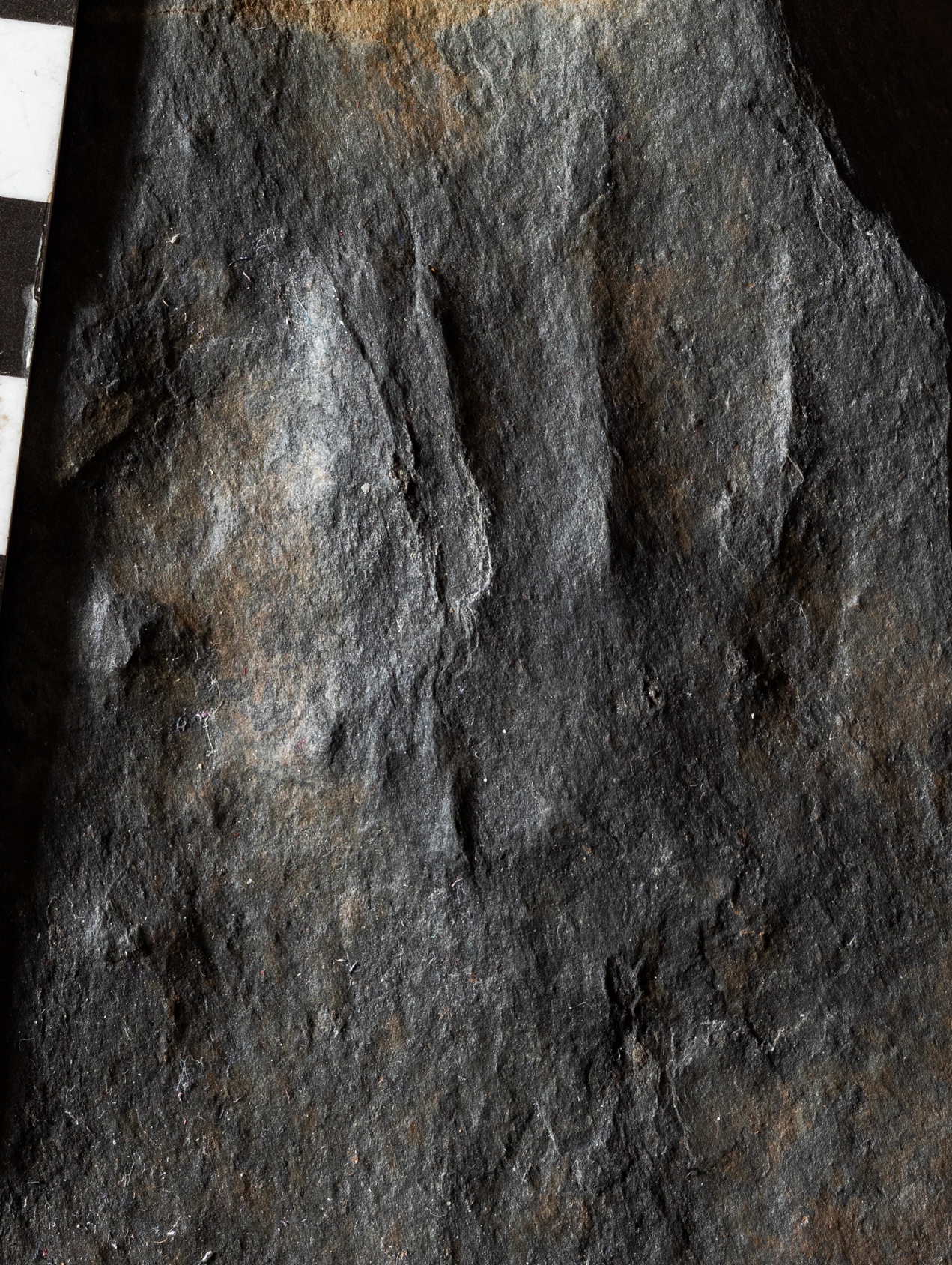


Supplementary Figure 15. IVPP V31909, an unidentified manus/pes imprint


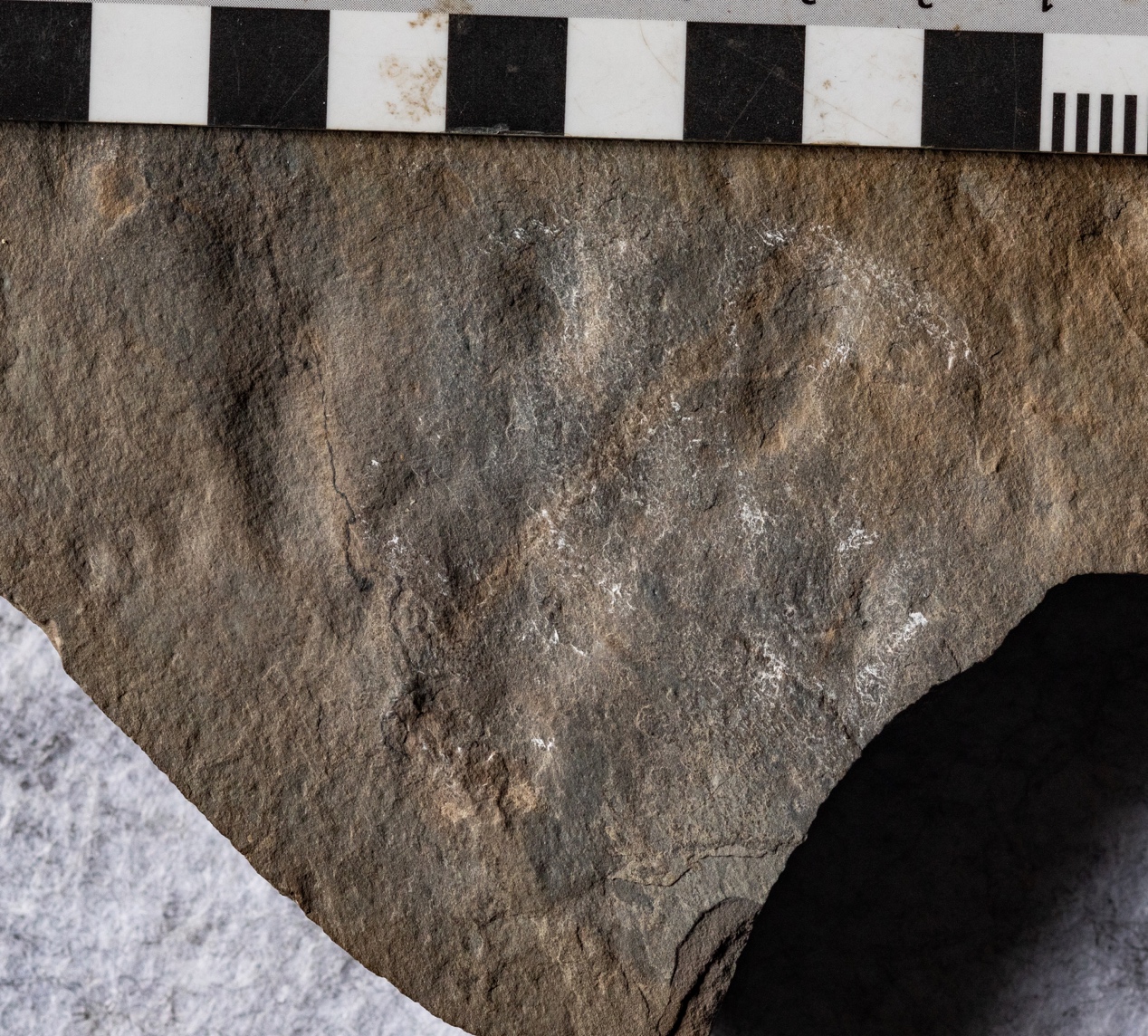


Supplementary Figure 16. An unidentified manus/pes imprint.


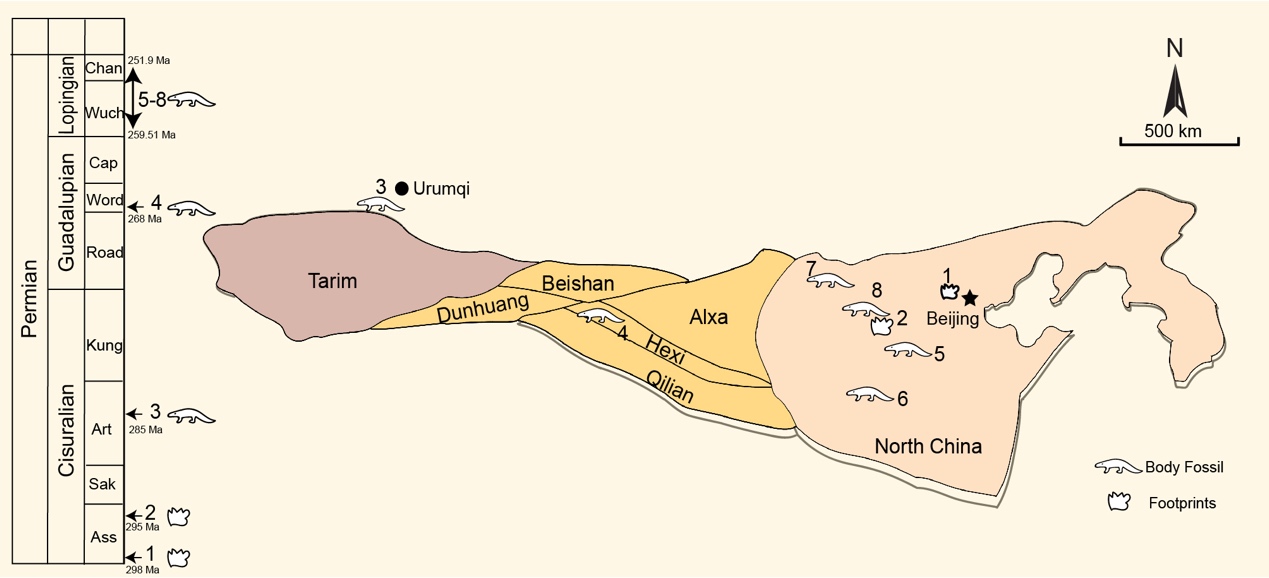


Supplementary Figure 17. Stratigraphic column and map of tetrapod-bearing localities in northern China. The numbers 1-8 represent tetrapod localities in northern China in the order from old to young (1, 2, and 5-8 belong to the North China Block; 3 belongs to the Kazakhstan Block; 4 belongs to the Hexi Block): 1. Mentougou Ichno-Fauna: Mentougou, Beijing (Taiyuan Formation, ~298 Ma); 2. Single possible *Dimetropus* footprint: Baode, Shanxi (Lower Shihhotse Formation, ~295 Ma); 3. *Urumqia liudaowanensis*: Urumqi, Xinjiang (Lucaogou Formation, ~285 Ma); 4. Dashankou Fauna: Yumen, Gansu (Qingtoushan Formation, ~268 Ma); 5-8. Dicynodont-pareiasaur Fauna: 5. Yangquan, Shanxi (Sunjiagou Formation, 259-252 Ma); 6. Jiyuan, Henan (Sunjiagou Formation, 259-252 Ma); 7. Baotou, Inner Mongolia (Naobaogou Formation, 259-252 Ma); 8. Erdos, Inner Mongolia (Sunjiagou Formation, 259-252 Ma).
